# Collecting standardised oral health data via mobile application: A proof of concept study in the Netherlands

**DOI:** 10.1371/journal.pone.0191385

**Published:** 2018-02-07

**Authors:** Joost C. L. den Boer, Ward van Dijk, Virginie Horn, Patrick Hescot, Josef J. M. Bruers

**Affiliations:** 1 Research & Information Department, Royal Dutch Dental Association (KNMT), Utrecht, the Netherlands; 2 FDI World Dental Federation, Geneva, Switzerland; 3 Academic Centre for Dentistry Amsterdam (ACTA), University of Amsterdam and VU University, Amsterdam, the Netherlands; National Taiwan University, TAIWAN

## Abstract

FDI World Dental Federation, founded as Fédération Dentaire Internationale, has taken the initiative to develop the Oral Health Observatory, a mobile application to conduct oral health surveys worldwide. The aim is to collect reliable standardized international data on oral health and oral health care via a network of dentists. A proof of concept study project was set up in the Netherlands to test the methodology and to validate the approach. Data about caries, gingivitis, oral self-care and oral health related quality of life were analysed and compared to datasets validated in other studies. The Android app embeds three questionnaires addressing oral health history, status and patient behaviour. One questionnaire was completed by the patient and two by the dentist. The proof of concept study involved two phases: in the first phase, five dentists, regular participants in KNMT-surveys, evaluated the usability of the app; after the first phase, the app was adjusted for a second phase. For this phase an extra 15 dentists were recruited from a group of 20 other dentists: five of them declined to participate. Attention was paid to ensuring there was a proportional representation of gender, age and region. In the second phase the five first and 15 new participants collected data on up to a maximum of 38 patients. Data from this 653 patients correspond with results from previously published surveys on the prevalence of caries and gingivitis in the Netherlands. Hence demonstrating an association between caries and gingivitis with oral self-care, problems eating and experiencing oral pain. This proof of concept study shows that the app makes it possible to collect reliable information on oral health in a short period of time. Both dentists and patients evaluated the methodology as user-friendly. Altogether, the results of this proof of concept study are promising.

## Introduction

International comparisons on oral health, oral care by patients and care provided by oral health professionals are rare. Thus it appears, for example, that on the prevalence of caries ˗ which is the most widespread chronic disease worldwide and constitutes a major global public health challenge [1].

There is only a limited amount of reliable standardized data from different countries available [2][3]. Homogeneous data on periodontal problems are also absent due to the diversity of indicators used [4][5]. According to the FDI World Dental Federation (FDI), founded in 1900 under the name Fédération Dentaire Internationale, this is related to the fact that oral health does not get regular attention in national epidemiological surveys, whilst the execution of separate surveys on oral health is expensive and complex [1]. The lack of reliable data complicates the advocacy efforts of governments in developing policies to improve oral health. For example, in the Netherlands there is insufficient knowledge about the actual needs with regard to oral health [6].

In Vision 2020, FDI’s objectives for the future, FDI mentions the promotion of research into oral health across several countries [7]. Therefore, it is crucial to obtain international consent about what is generally understood by oral health and oral care and also about standardized methods for data collection and analysis within this field [8]. Ideally, this data could be retrieved from electronic health records (EHRs). But there still are many challenges concerning the deployment of EHRs and communication standards for EHRs [9][10]. The World Health Organization (WHO) recognizes the need for standardized data on oral health and has striven towards this for years [11][12]. A standard method for measuring oral health offers the possibility to compare data from different countries, even when this data has been collected in separate surveys.

In a move towards such a standardized research method, FDI has taken the initiative to develop the Oral Health Observatory (OHO) [13]. Via this new approach, FDI aims to collect comparable data on: oral health in children and adults from individual dental practices, in different countries; national (oral) health-care systems; and oral health in relation to the quality of life. The emphasis is put on the relation between caries and gingivitis on the one hand, and on aspects of oral care and the quality of life on the other. This choice found its rationale in two facts: (1) caries and gingivitis are endemic oral diseases and (2) aspects of lifestyle, such as dietary habits and oral care, are known as important risk factors in the disease aetiology [14][15]. For instance, it is recognized worldwide that tooth brushing is slowing down the development of caries and gingivitis [16]. In addition, a correlation between the burden of caries and a poor quality of life has been demonstrated [17][18]. The impact of gingivitis on quality of life has been proven to a lesser extent, or only marginally; but as for more serious periodontal diseases, which could lead to tooth loss, the connection is definitely apparent [19].

This paper presents the results and conclusions of an OHO proof of concept study project conducted in two phases in the Netherlands, which was part of a larger proof of concept project in three countries (Germany, Mexico and the Netherlands). The objective of this paper is to determine the research possibilities offered by the OHO to collect data on oral health and oral care in dental practices using electronic patient and dentist questionnaires on tablet computers. Firstly, the method of data collection is explained and then some basic results are described. Finally, findings are presented of the interviews with participating dentists about their assessment of the OHO-instrument.

## Materials and methods

### Measuring instrument

Three questionnaires have been developed for the OHO. Table 1 shows the questions posed per questionnaire. The first questionnaire is aimed at collecting background patient data and is completed by the dentist or a member of staff in the practice, before the patient concerned visits the practice (Table 1A). The second questionnaire concerns the patient’s oral health and the dentist completes this after the patient’s visit (Table 1B). Finally, the third questionnaire is completed by the patient and involves them providing background data on themselves and their oral health (Table 1C). In the case of younger patients, it was completed by a parent or carer. The third questionnaire, which is based on the WHO research method, can be completed before or after a visit to the dentist [12].

**Table 1:** Questions of the questionnaires of the proof of concept study of the oral health observatory.

**A**	**Background characteristics of patients, provided by the dentist or an employee of the dental practice before consultation**
A1	What is the gender of this patient?
A2	What is the age of this patient?
A3	Is this patient visiting you / your practice regularly (at least once a year)?
A4	What is the reason for this visit?
**B**	**Clinical evaluation of the patients’ oral health, provided by the dentist after consultation**
B1	Does this patient have cavitated caries?
B2	Does this patient have any restorations?
B3	Does this patient have any sealants present?
B4	Is there any tooth removed on this patient (except for orthodontic reasons)?
B5	Does this patient have gingivitis?
B6	Does this patient have bone loss?
B7	Does this patient use tobacco in any way (smoking/chewing)?
**C**	**Background characteristics and oral health, provided by the patient before or after consultation**
C1	What is the reason for this visit?
C2 ^#1^	Does your child go to school/nursery/preschool?
C3 ^#2^	Do you go to school?
C4 ^#3^	What school did you finish?
C5 ^#3^	Which was your main professional occupation during the past 12 months?
C6	Where do you live?
C7	When did your child last visit a dentist?
C8	How often did you/your child visit the dentist in the last year?
C9	What was the reason for the last visit (not this one)?
C10	What was the main reason why you/your child did not visit the dentist in the last year?
C11	If you/your child see the dentist/dental practice, how long is your travel either from school, work or home?
C12	If you / your child needs dental care, do you usually get an appointment easily?
C13	If needed, I can get an appointment (within) … … …
C14	Do you/ does your child smoke or chew any form of tobacco?
C15	If, yes. How often?
C16	How many eating/drinking occasions, do you/does your child have per day (including snacks, small quantities like candy, coffee, tea, soft drinks etc.)?
C17 ^#4^	Is your child still bottle/breast- feeding?
C18	How often have you experienced difficulties with eating food due to mouth and teeth problems?
C19	How often have you experienced toothache/painful gums/sore spots?
C20 ^#4^	How often was your child unable to go to the nursery / preschool due to mouth or teeth problems?
C21 ^#5^	How often have you felt embarrassed because of the appearance of your teeth [or dentures]?
C22 ^#5^	Have you avoided smiling / laughing because of the appearance of your teeth [or dentures]?
C23 ^#6^	How often were you unable to go to work or school due to mouth or teeth problems?
C24	How often have you reduced your participation in social activities because of problems with mouth or teeth?
C25	How often do you brush your teeth?
C26	When do you brush your teeth?
C27	Do you use a toothpaste containing fluoride?
C28	Do you use any of the following fluoride products other than toothpaste?

#1. only patients of 11 years and younger.

#2. only patients from 12 up to and including 17 years.

#3. only patients of 18 years and older.

#4. only patients of 5 years and younger.

#5. only patients of 12 years and older.

#6. only patients of 6 years and older.

These three questionnaires were developed into a mobile application (app). By creating a specific app, the changes of misuse of the data are minimized. The app can be used on tablets with an Android operating system: Samsung Galaxy Tab 4.10.1 (model number SM-T530 and Android version 5.0.2). The questionnaires were completed in the practice with or without access to the internet. Synchronization of the data with the centralized database could be done in real-time, upon internet access availability. In circumstances where there was no network connection, all data were stored until they could be synchronized when a network connection was available. Since the three questionnaires concerning an individual patient are linked to each other, a cross analysis can be made between the background characteristics of the patient, their oral care behaviour and well-being, and the clinical status reported by the dentist.

### Ethics statement

Dentists who consented to take part in the study were visited and instructed how to recruit patients for the study. The purpose of the study and the nature of participation was introduced to potentially participating patients and parents of young patients at a regular dentist visit. After this introduction they were asked to participate. Those who consented participation completed a questionnaire and gave the dentist permission to fill out two questionnaires about their (child’s) background and oral health status. This study did not contain any other interventions or additional examinations. The study protocol was approved by an independent review board of the KNMT.

### Set-up of the first phase of the proof of concept study

The OHO proof of concept study has been conducted in two phases. Five dental practices in the Netherlands participated in the first phase, which took place in September 2014. The Netherlands was a suitable country for this purpose since dental practices in this country are familiar with surveys conducted by the dental professional organization. As it happens, the Royal Dutch Dental Association (KNMT) has had its own research programme for 20 years which focuses on periodic data collection in dental practices about practising the dental profession [20][21]. Five dental practices selected from the participants in KNMT-surveys, were initially approached by telephone. All five practices that agreed participation and were visited for the delivery of a tablet with the OHO app installed and given oral instructions on how to use it. During the first phase of the proof of concept study, the participating practices were in regular phone or email contact with the KNMT-office about how to collect the data.

In the first phase a questionnaire in English was used as translating the questionnaire would have been a timely process. Besides, the intention was not so much focused on collecting usable data on oral health and the patients at this stage, but rather to test whether or not data collection via an app on a tablet in the dental practice would work and could be reliable. In addition, it is reported that a large majority of Dutch people say they have sufficient command of the English language [22]. In the five practices, data was collected via the app for a maximum of about 10 patients from various age groups. The collected data was sent from the tablet via the internet to a database managed by FDI. Patients remained anonymous in the database. Dentists could do this at any given moment, for example immediately after the patient consultation, at the end of each day, or after seeing all the patients.

Both dentists and patients were enthusiastic about this method of data collection. Some questions, however, still proved to be somewhat unclear and the dentists found that the app reacted slowly. The data collected in this first phase has not been used in the results as described below.

### Set-up of the second phase of the proof of concept study

Where possible, the comments received in the first phase were incorporated into the questionnaires and the app. After this the questionnaires were translated into German, Spanish and Dutch for the second phase of the proof of concept study, which was conducted more or less simultaneously in Germany, Mexico and the Netherlands. In each of these three countries, 20 dentists were prepared to participate in the second phase. The second phase started in the Netherlands at the end of March 2015 and lasted until mid-2015. In the Netherlands the five dentists who had already participated in the first phase took part. Following the same procedure as used in the first phase, 15 other dentists were recruited from a group of 20 dentists, five dentist refused participation. During the selection process, attention was paid to ensuring there was a proportional representation of gender, age and region.

The 20 dentists were first sent an online questionnaire in order to collect some general data about their practice organization. Subsequently, each one of them was visited in person to deliver the tablet and explain first-hand how to use it. All the information and instructions were also made available in writing, partly in English and partly in Dutch. On the basis of this material one of the dentists made an information and instruction poster for the patients. This was subsequently offered by the KNMT to several of his colleagues and was thus used in more practices.

The dentists were asked to collect data from a total of 38 patients via the OHO app: six patients, three male and three female, from each age group of youths (ages: 2-≤5 years, 6-≤11 years and 12-≤17 years), and 20 adult patients (aged ≥18 years), 10 male and 10 female. As in the first phase of thestudy, the patients remained anonymous. When selecting the patients, dentists were asked to pay attention to securing an even spread of age and gender.

### Statistical analysis

From the FDI database the data collected from the Netherlands was made available for further analysis to the KNMT via an Excel file. After conversion of this file the data was analysed with the aid of IBM SPSS Statistics, version 20. Thus, an overview of frequencies and means was first obtained from the straight counts, in which a distinction was made as regards age. Subsequently, for two aspects of oral health, viz. the presence of visually detectable caries and gingivitis, the bivariate cohesion with some other characteristics was shown (Chi-quadrate test, t-test, ANOVA).

### Assessment of the proof of concept study by dentists

During the course of the proof of concept study there was regular contact, by telephone and e-mail, on the survey’s progress between the researchers and the participating dentists. During such contacts interviews were conducted on matters such as app user-friendliness, general experiences or any other issues the dentists or patients had were discussed.

## Results

From the practices of the 20 participating dentists, useful data was collected from a total of 653 patients. This amounts to 86% of the total number of patients from whom information could have been potentially collected. Twelve dentists supplied data on the maximum number of 38 patients, whereas eight dentists were not able to do this. In their cases the numbers of patients varied from eight up to and including 37. Table 2 shows that the intended representation as regards gender and age was realised. Furthermore, it is highlighted that a majority of the patients were regular attendants of the practices concerned.

**Table 2:** Background characteristics of patients, provided by the dentist or an employee of the dental practice.

**Sex**	
Male	49%
Female	51%
Unknown	%
**Age**	
3–5 years	14%
6–11 years	15%
12–17 years	15%
18 years or older	56%
**regular visiting patient**	
No	6%
Yes	94%
**reason for this visit**	
check-up	65%
routine treatment	28%
emergency treatment	7%
Unknown	%
n = 653	

### Oral health

The survey results as shown in Table 3, demonstrate that dentists visually detected cavitated caries after the consultation in 16% of the patients and that in 63% they saw one or more oral restorations. Furthermore, the dentists noted in 40% of the patients that at least one tooth had been extracted. In 27% they saw sealants and in 32% there was gingivitis. Bone loss was detected in 19% of the patients and according to the dentists 10% turned out to be smokers. Furthermore, it appeared that virtually all the characteristics of oral health were age-related.

**Table 3:** Clinical evaluation of the patients’ oral health, provided by the dentist.

		*2–5 years*	*6–11 years*	*12–17 years*	*18+ years*	total
*Patients with*:						
- restorations	*	9%	37%	42%	90%	63%
- teeth/tooth removed (except for orthodontic reasons)	*	2%	12%	14%	63%	40%
- gingivitis	*	1%	16%	32%	43%	32%
- sealants	*	5%	53%	75%	14%	27%
- bone loss	*				33%	19%
- cavitated caries		11%	22%	14%	17%	16%
- smoking habit	*			8%	16%	10%
n		92	99	96	366	653

* differences between age-groups are statistically significant (p < 0,01).

### Background characteristics and oral health according to the patients themselves

As is shown in Table 4, the vast majority of the patients in the survey (92%) had also seen their dentist in the year prior to this present visit. A total 84% of the patients also stated that they brushed their teeth at least twice a day. With regards the questions concerning the influence of oral problems on their functioning, only a small minority (15% or less) stated that they faced this regularly.

**Table 4:** Results of the OHO-questionnaire about background characteristics and oral health, provided by the patient.

		*2–5 years*	*6–11 years*	*12–17 years*	*18+ years*	total
**day-time occupation**	*					
nursery, preschool or school		46%	97%	98%	5%	39%
work (self-employed or employed for wages)					74%	41%
no nursery, (pre)school or work		54%	3%	2%	20%	20%
**highest finished education**						
no school		^**#1**^	^**#1**^	^**#1**^	%	%
elementary school		^**#1**^	^**#1**^	^**#1**^	2%	2%
college school or comparable		^**#1**^	^**#1**^	^**#1**^	64%	64%
high school, bachelor degree or university degree		^**#1**^	^**#1**^	^**#1**^	34%	34%
**urbanisation rate municipality**						
< = 3.000 habitants		9%	7%	10%	12%	11%
3.001–15.000 habitants		48%	54%	58%	45%	49%
> = 15.001 habitants		43%	38%	32%	43%	40%
travelling time to dentist less than 30 minutes		96%	95%	91%	89%	91%
easy to get an appointment with the dentist		100%	100%	99%	99%	100%
possible to get appointment with dentist within 24 hours	*	83%	67%	57%	62%	65%
visited dentist in the last year	*	83%	97%	98%	92%	92%
number of visits to dentist in the last year (only visitors)		1,8	2,0	2,3	2,3	2,2
smokes	*			8%	20%	13%
number of eating/drinking occasions per day		6,2	5,4	6,1	5,7	5,8
**tooth brushing frequency**	*					
never		4%	3%	1%		1%
once a week				1%	1%	1%
a few times a week			1%	2%	1%	1%
once a day		9%	15%	4%	15%	13%
twice or more a day		87%	81%	92%	83%	84%
using a toothpaste containing fluoride		95%	99%	93%	95%	95%
consequences of mouth and teeth problems						
occasionally or very often problems eating	*	1%	7%	7%	12%	9%
occasionally or very often toothache/painful gums/sore spots	*	3%	12%	13%	19%	15%
occasionally or very often felt embarrassed		^**#1**^	^**#1**^	8%	14%	13%
occasionally or very often avoided smiling/laughing		^**#1**^	^**#1**^	7%	9%	8%
occasionally or very often unable to go to nursery/(pre)school/work			1%		1%	1%
occasionally or very often avoided participation in social activities					%	%
n = 89–653						

#1. no data available for this age-group.

* differences between age-groups are statistically significant (p < 0,01).

### Cohesion with caries and gingivitis

Table 5 shows that in the survey patients in whom cavitated caries were visually detected by the dentist, they had on average more eating and drinking moments per day than those in whom cavitated caries had not been visually detected (6.4 versus 5.7). Furthermore, a smaller proportion of this group brushed their teeth at least twice a day (70% versus 87%), a larger proportion had eating problems due to issues in or with their mouth (19% versus 7%) and a larger proportion of patients also had problems with aching teeth, molars and/or painful gums (27% against 12%).

**Table 5:** Aspects of oral self-care and oral heath related quality of life in cohesion with the presence of cavitated caries.

		no cavitated caries			cavitated caries			total
		< 18 years	> = 18 years	total	< 18 years	> = 18 years	total	
number of eating/drinking occasions per day	*	5,8	5,5	5,7	6,5	6,4	6,4	5,8
brushing teeth twice or more a day	*	89%	85%	88%	72%	68%	70%	84%
using a toothpaste containing fluoride		96%	95%	96%	95%	92%	94%	95%
occasionally or very often problems eating	*	3%	11%	7%	18%	19%	19%	9%
occasionally or very often toothache/painful gums/sore spots	*	7%	17%	12%	25%	28%	27%	15%
occasionally or very often felt embarrassed		8%	13%	12%	8%	19%	17%	13%
occasionally or very often avoided smiling/laughing		7%	7%	7%	8%	16%	15%	8%
occasionally or very often unable to go to nursery/(pre)school/work			1%	1%	3%	2%	2%	1%
occasionally or very often avoided participation in social activities			1%	0%				0%
n = 89–649								

* differences between groups with and without cavitated caries are statistically significant (p < 0,01).

In Table 6 the same aspects of oral care, behaviour, and the results of oral problems have been linked to gingivitis whose presence had been detected by the dentist. This shows that patients with gingivitis, in comparison with patients without, brushed their teeth at least twice a day in fewer cases (78% versus 87%). Moreover, a larger proportion had eating problems resulting from issues in, or with their mouth (17% versus 5%) and a larger proportion also had aching teeth, molars and/or painful gums (24% versus 10%).

**Table 6:** Aspects of oral self-care and oral heath related quality of life in cohesion with the presence of gingivitis.

		no gingivitis			gingivitis			total
		< 18 years	> = 18 years	total	< 18 years	> = 18 years	total	
number of eating/drinking occasions per day	*	6,0	5,8	5,9	5,5	5,6	5,6	5,8
brushing teeth twice or more a day	*	88%	86%	87%	77%	78%	78%	84%
using a toothpaste containing fluoride		96%	95%	95%	95%	95%	95%	95%
occasionally or very often problems eating	*	2%	9%	5%	21%	16%	17%	9%
occasionally or very often toothache/painful gums/sore spots	*	6%	15%	10%	29%	23%	24%	15%
occasionally or very often felt embarrassed		11%	13%	12%	3%	16%	14%	13%
occasionally or very often avoided smiling/laughing		8%	6%	7%	7%	11%	11%	8%
occasionally or very often unable to go to nursery/(pre)school/work			1%	0%	2%	1%	1%	1%
occasionally or very often avoided participation in social activities			0%	0%		1%	0%	0%
n = 89–649								

* differences between groups with and without cavitated caries are statistically significant (p < 0,01).

### Assessment of the proof of concept study by dentists

The dentists in this study were very positive about answering the questions through the app and appreciated the ability to do this jointly during the patient’s visit to the practice. According to them also patients were positive about the OHO instrument. Furthermore, the dentists found that the instructions for the instalment of the app were good and they also valued the oral instructions they received though the direct contact hence there was no criticism on this side. The app was, however, found to be slow and was lacking visual response to input. It was recommended to increase the app reactivity. In addition, several dentists said that parents of children aged 6–11 years thought it strange they should be asked the question whether or not their child smokes. The suggestion that this was possible was sometimes considered to be insulting.

## Discussion

The OHO is intended to be a standard method for measuring aspects of oral health and oral care from an international perspective and is earmarked for making general comparisons between countries. This method is specific in that it brings together the perspectives of both the dentists and patients by using digital media. In addition, it also offers the possibility to set off the assessments of dentists against those of their patients. This is not unique, but the set-up of the OHO makes collecting such data relatively easy by using the network of dental practices [23][24]. This method offers advantages, but there are also drawbacks.

### Advantages

In this proof of concept pilot, the testing of the research methodology of the OHO was central as was the collection of reliable data. A comparison between the results derived from this study and the results from other surveys shows similarities. Thus, the outcomes with regard to brushing teeth can be compared to those of earlier research in the Netherlands [25][26]. The results with regards to dental visits show a slight over estimation, but this can be explained by the fact that this proof of concept study was conducted in dental practices, resulting in an automatic over-representation of dental visitors [27]. The internationally recognized relation between brushing teeth and caries and periodontal aberrations was also confirmed [16]. The effect of fluoride toothpaste cannot be proven in this study, since nearly all patients in the Netherlands are using such toothpaste. It is true that for the collection of clinical data an independent and calibrated oral inspection is preferred, but doing this is very time and labour consuming [28]. The set-up of the OHO makes the oral inspection for the collection of clinical data less time consuming and in comparison takes no extra effort, making it less costly. After all, the dentist will in any case perform an oral inspection with patients who come for treatment or a regular dental check-up. A precondition is, however, that the intention is to make a global assessment of oral health, on the basis of aspects that can be determined as objectively as possible and must also be visible to the naked eye.

In the Netherlands finding 20 dentists across the country, who were prepared to take part in the proof of concept study, proved to be easy. In addition, these dentists generally had little difficulty in recruiting patients for the survey. It did appear to be important that the researchers remained in close contact with the dentists in the study by means of a visit to the practice, oral instructions and regular contact. This limited any uncertainties and gave dentists the feeling that they were not ‘left to their own devices’ in case of problems. Perhaps an environmental advantage was that dentists in the Netherlands are used to research being conducted by the national professional organization [20].

Collecting data on a tablet by means of an app is done relatively quickly because the information is directly sent to a central database. In a systematic literature study of different ways of recording Patient-Reported Outcome Measures, no differences in results between questionnaires submitted in writing, or electronically (for example via tablets) were found [29]. The collection of data via an app may therefore, be considered to be reliable.

### Challenges

The difficulty with international comparisons is that all kinds of national circumstances can influence a phenomenon to be measured. This will also apply when using an app. Thus, for example, it appeared during the study that parents of young children were sometimes irritated by the question whether or not their child was smoking. However, in some countries this may be a relevant question. In short, compiling a questionnaire which should be applicable for each country is not easy due to such cultural and social influences. Thus, certain questions may be subject to interpretation depending on the survey country. Take for example the travel time to the practice as an indicator of access to dental care. From the study it appeared that in the Netherlands 9% of patients had to travel for more than half an hour to the dental practice. However, the Netherlands is a densely populated country with one dentist in approximately every 1,900 inhabitants [30][31]. If a patient has to travel for more than half an hour to go to the dentist, then this is relatively far for the Netherlands and possibly a deliberate choice. In a different country travelling for half an hour may mean a relatively short distance. Therefore, one has to be alert and choose the right context when interpreting the outcomes.

Although collecting clinical data within the OHO by the dentist offers practical advantages there are also reservations. A first concern is that there may be biases present in that only those who visit the dentist are involved in the survey. This does not generate a complete picture, as the oral health of non-attendants is therefore not taken into account. In the Netherlands this distortion can be considered minor, as a vast majority of the population visits a dentist [32]. But we certainly speak of a distortion, which will be greater in countries with fewer dental visits.

A second point concerns the multi-level character of the data to be collected [33].

Within the OHO the collection of patient data takes place within dental practices. Therefore, the measurements at a patient level are not automatically independent, but dependent on the dentist and/or the practice. The average number of restorations per patient, for example, may differ as a result of variations in treatment [34].

The app developed for the OHO is undeniably attractive to participants in the survey. But then its fast and accurate functionality is indeed a precondition. Especially, since the speed of the app sometimes proved to be a problem in this survey.

### Recommendations

In August 2016, FDI announced the deployment phase of the OHO [35]. From the proof of concept study in the Netherlands it was highlighted that the OHO is a practical instrument to gain general insight from dental practices into the oral health and oral care of a particular country, specifically with a view to make international comparisons between countries. To make this possible a standardized and valid questionnaire containing is required. The questionnaire used in this study did not meet this criteria. Furthermore, as far as content is concerned this instrument has its limitations since only those who visit the dentist are involved. With respect to the methodology the bias that can occur because of social differences between countries should be taken into account. Bias, since dentists ‘score’ their own care and in connection with this the multi-level character of the clinical data [36]. In addition, it is advisable to further develop the OHO app. A new version of the app should be faster, more user-friendly and more flexible. As for the latter point it is of particular importance to National Dental Associations (NDA’s) that they themselves, or in collaboration with stakeholders, have the possibility to add their own questions about specific national issues (e.g. man power planning and quality policies). Thus FDI offers its members a project that can be deployed not just internationally, but also nationally. This may have a positive influence on an NDA’s willingness to participate in the OHO.

There is no doubt that the proof of concept study in the Netherlands has proven that the OHO can be used well, and there is also great value in the information content the instrument offers. The extent however, to which this information can be compared in an acceptable way to the information collected via the OHO in other countries needs further investigation. A further analysis of the Dutch data in relation to the data collected in Germany and Mexico offers every possibility to do so. This could be the next step in the process to achieve, in time, an international comparison between countries on oral health and oral care, which is content relevant and methodologically reliable. The FDI initiative of the OHO offers a very promising perspective.
